# Physical Performance, Muscle Strength, and Frailty: Differential Associations with Health-Related Quality of Life in Peri-Urban Older Adults

**DOI:** 10.3390/healthcare14213625

**Published:** 2026-10-26

**Authors:** Leydi Ordoñez, Yoconda Arias, Cindy Carriazo, Maura Pérez, Miguel Terán, Fermina Vasquez Osorio, Adelaida Zappa, Luz Villadiego

**Affiliations:** 1 Health and Movement Research Group, Faculty of Health, Universidad Santiago de Cali, Santiago de Cali 760035, Colombia; leidy.ordonez01@usc.edu.co; 2 Programa de Fisioterapia, Universidad del Sinú, Montería 230001, Colombia; yocondaarias@unisinu.edu.co (Y.A.); cindycarriazo@unisinu.edu.co (C.C.); mauraperez@unisinu.edu.co (M.P.); ferminavasquez@unisinu.edu.co (F.V.O.); adelaidazappa@unisinu.edu.co (A.Z.); luzvilladiegod@unisinu.edu.co (L.V.)

**Keywords:** older adults, frailty, muscle strength, physical performance, health-related quality of life

## Abstract

**Highlights:**

**What are the main findings?**

- A high prevalence of pre-frailty (82.6%) was observed among the older adults included in this study.
- Grip strength was independently and positively associated with the physical functioning domain of the SF-36.
- Grip strength did not show an independent association with the study-specific mean of the SF-36 domain scores.
- Physical performance assessed using the SPPB showed no independent association with either the study-specific mean of the SF-36 domain scores or the physical function domain.
- A higher study-specific mean of the SF-36 domain scores was associated with frailty; however, these results should be interpreted with caution due to the small number of participants in the robust and frail categories.

**What are the implications of the main findings?**

- Health-related quality of life (SF-36): Its variation is multifactorial, meaning that a physical variable may be associated with greater or lesser intensity across the domains.
- The specific relationship between handgrip strength and perceived physical function.

**Abstract:**

**Introduction**: Population aging highlights the need to identify functional factors related to the health and quality of life of older adults. Frailty, physical performance, and muscle strength are relevant indicators of functional status, although their relationship with health-related quality of life (HRQoL) may vary depending on the component being assessed. **Objective**: To analyze the differential associations between physical performance, muscle strength, and frailty status with health-related quality of life in older adults in Montería, Colombia. **Methods**: An observational, cross-sectional study was conducted with 155 adults aged 60 years and older. Physical performance was assessed using the Short Physical Performance Battery (SPPB), muscle strength using a handgrip dynamometer, and frailty using the Fried phenotype. Quality of life was assessed using the Short Form-36 Health Survey (SF-36). Correlation analyses, multiple linear regression, and multinomial logistic regression were performed, adjusted for sociodemographic and clinical variables. **Results**: The mean age was 71.94 years, and 81.9% of the participants were women. Pre-frailty had a prevalence of 82.6%, while 9.0% were classified as robust and 8.4% as frail. Handgrip strength and the SPPB were not significantly associated with the study-specific mean of the SF-36 domain scores. However, handgrip strength showed an independent association with the physical functioning domain. Furthermore, a higher study-specific mean of the SF-36 domain scores was associated with a higher probability of being in the robust group and a lower probability of frailty. **Conclusions**: Quality of life is differentially associated with muscle strength and frailty status. Early identification of pre-frailty and a comprehensive assessment of physical function can help guide preventive strategies in older adults.

## 1. Introduction

When we talk about aging, it is important to understand that this population represents one of the most significant demographic phenomena of the 21st century. The number of people aged 60 and older is projected to double worldwide between 2020 and 2050, rising from 1 billion to 2.1 billion, while their share of the total population will increase from 12% to 22% over the same period [1]. This demographic shift, recognized by the World Health Organization through the Decade of Healthy Aging 2021–2030, highlights the urgent need to understand the functional determinants that influence active and independent aging [2].

Frailty is recognized as a clinical syndrome associated with a decline in physiological reserve and a reduced ability of the body to respond adequately to various stressors. One of the most commonly used methods for identifying frailty is the phenotype proposed by Fried et al., which considers five criteria: unintentional weight loss, exhaustion, decreased walking speed, muscle weakness, and low physical activity levels [3]. The presence of frailty is of particular importance in this population due to its association with adverse outcomes such as falls, disability, hospitalization, and an increased risk of mortality [3]. In Latin America and the Caribbean, prevalence rates ranging from 7.7% to 42.6% have been reported, with estimates suggesting that approximately one in five older adults has this condition [4]. In Colombia, the SABE study reported a national prevalence of frailty of 17.9%, and its frequency varies according to characteristics such as age, sex, and sociodemographic conditions [5,6].

Frailty in older adults is associated with a decline in functional reserve and, therefore, with an increased risk of progressive health decline in this population. Evidence suggests that frailty can change over time—it may improve, remain stable, or worsen—and that these changes are associated with health-related quality of life. Likewise, those who maintain or improve their frailty status have shown better outcomes in the physical and social domains, while worsening frailty has been linked to a decline in quality of life [7]. Therefore, frailty, in addition to being a clinical risk marker for this population, also reflects how people perceive their functioning and well-being.

Likewise, physical performance and muscle strength are considered key factors that may explain the link between frailty and quality of life. In a study of older adults that implemented a multicomponent intervention, the program was associated with improved physical performance and muscle strength in the upper limbs and trunk, as well as an increase in perceived quality of life [8]. Furthermore, evidence from studies focusing on exercise interventions in older adults with frailty shows that components such as strength and endurance improve physical function, which is key to maintaining independence and improving quality of life [9,10].

In this context, the assessment of functional capacity is an important component in understanding the overall health status of older adults. To this end, objective tools such as the Short Physical Performance Battery (SPPB), handgrip strength testing, and the Short Form-36 Health Survey are used. Handgrip strength provides insight into muscle function and can reflect changes associated with the aging process, including the loss of motor units, fat infiltration in muscle tissue, and various neuroendocrine changes. A decline in handgrip strength has been linked to an increased risk of adverse health outcomes [11]. Complementarily, the SPPB assesses three fundamental components of physical performance: balance, walking speed, and the ability to repeatedly stand up from a chair. This battery has proven useful for identifying functional impairments and predicting outcomes such as disability, falls, and mortality in older adults [12].

Assessing health-related quality of life (HRQoL) is essential in this population group, and one of the most widely used instruments for this purpose is the 36-Item Short Form Health Survey (SF-36), which assesses multiple dimensions of perceived physical, social, and emotional health [13]. However, the available evidence on the relationship between HRQoL and physical performance is mixed. Some studies show a nonlinear association between handgrip strength and overall quality of life [14], while others report sex-specific differences in the relationship between handgrip strength and HRQoL [15]. A recent scoping review further indicates that differences in measurement methods and outcome definitions limit comparability across studies examining handgrip strength and health-related outcomes [11]. Frailty has also been associated with poorer health-related quality of life across physical and mental dimensions [16].

On the other hand, falls are events frequently linked to frailty and impairments in physical performance, and a significant association between a history of falls and poorer quality of life among older adults has been demonstrated [17]. However, there remains a gap in our understanding of the correlation between objective measures of overall physical performance, handgrip strength, physical function, and frailty—and how these factors impact quality of life across specific domains—particularly among older adults in Latin America, where evidence remains limited compared with that available for European and Asian populations [4,5].

Given these gaps in knowledge, this study aims to analyze the differential associations between physical performance, muscle strength, and frailty status with health-related quality of life in a sample of older adults from the city of Montería, Colombia.

## 2. Materials and Methods

### 2.1. Study Design

An observational, cross-sectional study was conducted among older adults living in Montería, Córdoba, Colombia. Data were collected between July 2025 and July 2026. The aim was to examine the relationship between physical performance, muscle strength, frailty, and health-related quality of life. This study was reported in accordance with the STROBE guidelines for cross-sectional studies [18].

### 2.2. Population and Sample

The population consisted of older adults living in peri-urban areas of Montería. The sample comprised 155 participants, selected by convenience sampling without probability sampling. Individuals aged 60 years or older who were able to understand the instructions for completing the questionnaires and performing the study’s functional tests were included. Participants were asked to sign an informed consent form to participate. Participants who were unable to safely undergo the assessments or who had incomplete data for the primary analyses were excluded.

### 2.3. Study Variables

Sociodemographic, anthropometric, clinical, and functional data were collected. Frailty, history of falls, and health-related quality of life were assessed. The sociodemographic variables were age, sex, and educational level. Age was treated as a continuous variable; sex and educational level were recorded as categorical variables. Body mass index (BMI) was calculated by dividing weight in kilograms by height in meters squared and was used as a continuous variable in the analyses. The burden of morbidity was classified as low if there were fewer than two comorbidities and high if there were two or more. Polypharmacy was defined as present when the individual was taking five or more medications per day.

All questionnaire-based instruments were administered in Spanish to ensure that participants could understand them. For instruments with validated versions for the Colombian population, those versions were used, and references to their adaptation and validation processes were included in the description of each instrument. In the case of physical performance tests and objective measurements, the standardized procedures described in their original protocols or in the versions used with the Colombian population were followed, as appropriate.

Assessment of Physical Performance: Physical performance was measured using the Short Physical Performance Battery (SPPB). The SPPB has three components: static balance, walking speed, and the ability to stand up from a chair multiple times. The score for each component was recorded according to the protocol and then added together to yield a total score ranging from 0 to 12 points. A higher score indicates better physical performance. In the primary analyses, the total SPPB score was used as a continuous variable.

### 2.4. Assessment of Frailty

Frailty was assessed using an adaptation of the physical phenotype proposed by Fried et al. [3], which comprises five components: weight loss, exhaustion, slow gait, muscle weakness, and low physical activity. The original criteria for assessing exhaustion, slow gait, and muscle weakness were retained, including sex-, height-, and body mass index-specific cutoffs, as appropriate. Adaptations were made to the operationalization of the weight loss and physical activity components, which are described in detail below. Each component was scored dichotomously, with one point assigned when the criterion was present and zero when it was absent. The total score ranged from 0 to 5 points; participants with 0 criteria were classified as robust, those with 1–2 criteria as pre-frail, and those with ≥3 criteria as frail.

Weight loss was assessed based on participants’ self-reports, with a loss of more than 3 kg during the three months prior to the assessment considered a positive criterion. This procedure is an adaptation of the weight loss criterion from the original phenotype described by Fried et al. [3], as both the reference period and the threshold used to identify this component were modified.

Exhaustion was assessed using the two questions from the Center for Epidemiologic Studies Depression Scale (CES-D) used in the original phenotype by Fried et al. [3]: “I felt that everything I did required a great deal of effort” and “I could not get started.” For each question, participants indicated how often they had experienced that feeling during the previous week. The exhaustion criterion was considered positive when the participant answered “a moderate amount of time (3–4 days)” or “most of the time” to either of the two questions, in accordance with Fried’s operationalization of the phenotype.

Walking speed was assessed based on the time required to walk 4.5 m at a normal pace. Classification was performed using specific cutoff points based on sex and height, in accordance with the phenotype described by Fried et al. [3]. In men with a height ≤ 173 cm, the criterion for slow walking was considered positive when the time was ≥7 s, while in those with a height > 173 cm, it was considered positive with a time of ≥6 s. For women with a height of ≤159 cm, a time of ≥7 s was considered slow walking, while for those with a height of >159 cm, the cutoff was ≥6 s. The 4.5 m distance used corresponds approximately to the 15 feet (4.57 m) employed in the original operationalization of the phenotype.

Muscle strength was assessed using a handgrip dynamometer. Before the test, participants received standardized instructions on the procedure. Participants were instructed to perform a maximal handgrip contraction for approximately 3 s. Two attempts were made with each hand, with a 1 min rest interval between attempts, and the highest recorded value was used for analysis. Muscle weakness was defined according to the grip strength cutoff points specific to sex and body mass index (BMI) described by Fried et al. [3]. For men, the cutoffs were ≤29 kg for a BMI ≤24 kg/m^2^, ≤30 kg for a BMI between 24.1 and 26 kg/m^2^, ≤30 kg for a BMI between 26.1 and 28 kg/m^2^, and ≤32 kg for a BMI >28 kg/m^2^. For women, the corresponding cutoffs were ≤17 kg for a BMI ≤23 kg/m^2^, ≤17.3 kg for a BMI between 23.1 and 26 kg/m^2^, ≤18 kg for a BMI between 26.1 and 29 kg/m^2^, and ≤21 kg for a BMI >29 kg/m^2^. Participants with grip strength values equal to or below the corresponding cutoff point based on sex and BMI were classified as positive for the weakness criterion of the frailty phenotype.

Physical activity level was assessed using the Reuben Hierarchical Questionnaire. Participants who reported not engaging in regular physical exercise or habitual walking were classified as meeting the criterion for low physical activity. This procedure represents an adaptation of the physical activity component of the original phenotype by Fried et al. [3], in which low physical activity is determined based on weekly energy expenditure, with cutoff points of <383 kcal/week for men and <270 kcal/week for women. Because a different instrument was used in the present study and weekly energy expenditure was not quantified using the original procedure, this component was considered a methodological adaptation of Fried’s criterion.

### 2.5. History of Falls; Occurrence of Falls

Each participant was asked whether they had experienced any falls in the 12 months prior to the evaluation. The responses were divided into two groups: no falls, or one or more falls during the past year.

### 2.6. Assessment of Health-Related Quality of Life

The 36-Item Short Form Health Survey (SF-36) was used. The SF-36 consists of 36 questions grouped into eight domains: physical functioning, physical role, bodily pain, general health, vitality, social functioning, emotional role, and mental health.

Scores for each of the eight SF-36 domains were transformed to a scale of 0 to 100, with higher scores indicating better perceived health. For the purposes of the present study, an additional study-specific composite measure was calculated as the arithmetic mean of the eight domain scores. This measure was used as a continuous variable in the statistical analyses. Because this arithmetic mean does not represent a validated standard SF-36 summary score, it is referred to throughout the manuscript as the “study-specific mean of the SF-36 domain scores” and should not be interpreted as equivalent to the standardized Physical Component Summary (PCS) or Mental Component Summary (MCS).

In addition to the overall measure, the individual scores for each area were retained. In particular, the physical function domain was analyzed due to its relationship with objective tests of physical performance.

### 2.7. Procedure

Once potential participants were identified, they were screened to ensure they met the selection criteria. Those who met the criteria were given an explanation of the study’s objective and the steps to be followed. Participation was voluntary, and informed consent was obtained before any assessment began.

Sociodemographic, anthropometric, and clinical information was then collected. Next, the instruments for assessing quality of life, frailty, and physical performance were administered. All assessments were conducted by physical therapists with experience in these procedures.

### 2.8. Statistical Analysis

The data were processed and analyzed using IBM SPSS Statistics, version 29 (IBM Corp., Armonk, NY, USA). In the first phase, the characteristics of the sample were described. Numerical variables were summarized using measures of central tendency and dispersion (mean, standard deviation, etc.). Categorical variables were presented as absolute frequencies and percentages. We examined how measures of physical function relate to quality of life using correlation coefficients. We looked at handgrip strength in the dominant hand and the total SPPB score and compared them with the study-specific mean of the SF-36 domain scores and its physical function domain.

Multiple linear regression models were then applied to determine whether objective measures of physical function were independently associated with health-related quality of life. The models were adjusted for age, sex, BMI, education level, multimorbidity, and polypharmacy. To interpret the results, the unstandardized regression coefficients (B), standardized coefficients (β), 95% confidence intervals, *p*-values, and changes in the coefficient of determination (ΔR^2^) were reviewed.

The association between quality of life and frailty status was examined using multinomial logistic regression. In the multinomial regression, frailty status was the dependent variable with three categories—robust, pre-frail, and frail—and the pre-frail category was used as the reference. The study-specific mean of the SF-36 domain scores was the primary explanatory variable, and the model was adjusted for age, BMI, and history of falls in the past year.

The results of the multinomial model were presented as adjusted odds ratios (aOR), 95% confidence intervals, and *p*-values. To facilitate clinical interpretation, the effect of the study-specific mean of the SF-36 domain scores was expressed per 10-point increase in this measure.

The overall fit of the model was assessed using the likelihood ratio test and the Cox-Snell, Nagelkerke, and McFadden pseudo-R^2^ values. The overall contribution of each variable was also analyzed using likelihood ratio tests.

Given the small number of participants classified as robust (*n* = 14) and frail (*n* = 13), compared with those classified as pre-frail (*n* = 128), the multinomial logistic regression analysis was considered exploratory. Therefore, the effect estimates were interpreted with caution, paying particular attention to the magnitude and width of the 95% confidence intervals.

As a sensitivity analysis, the total Fried phenotype score was analyzed using a generalized linear model with a Poisson distribution and a logarithmic link. The fit of the distribution was assessed using deviance and Pearson’s χ^2^ statistic compared to its degrees of freedom. Due to the observed underdispersion, this analysis was considered exploratory only and was not used as the primary inferential model. All statistical tests were conducted on a two-sided basis, and any *p*-value less than 0.05 was considered significant. When interpreting the results, the *p*-value, the magnitude of the estimators, and the 95% confidence intervals were taken into account.

### 2.9. Ethical Considerations

The research adhered to ethical principles for studies involving human subjects and complied with the guidelines of the Declaration of Helsinki. This study was approved by the Research Committee of the Faculty of Health Sciences at Universidad del Sinú (Institutional Committee Meeting No. 009 of 2026).

## 3. Results

A total of 155 older adults were included. The mean age was 71.94 ± 6.63 years, and the majority were women (81.9%). The mean BMI was 27.68 ± 4.57 kg/m^2^. 49.0% of the older adults had a high burden of morbidity, and 71.0% met the criteria for polypharmacy. In addition, 41 older adults (26.5%) reported at least one fall in the past 12 months. The sociodemographic, clinical, functional, and health-related quality-of-life characteristics of the participants are summarized in Table 1.

Regarding functional variables, the mean SPPB score was 7.23 ± 2.22 points, and the dominant handgrip strength was 20.08 ± 5.13 kg. The study-specific mean of the SF-36 domain scores was 57.02 ± 11.29 points, and the physical function domain had a mean of 50.04 ± 13.96 points. According to Fried’s phenotype, 14 older adults (9.0%) were classified as robust, 128 (82.6%) as pre-frail, and 13 (8.4%) as frail, showing a clear predominance of pre-frailty.

The high prevalence of pre-frailty observed (82.6%) should be interpreted in light of the operationalization used for the frailty phenotype. Although the components of exhaustion, slow walking speed, and muscle weakness adhered to the criteria established by Fried et al., the components of weight loss and physical activity were adapted to the procedures and instruments used in this study. These modifications may have influenced the distribution of participants across the categories of robustness, pre-frailty, and frailty and, therefore, may limit the direct comparability of the observed prevalence with studies that strictly applied the original operationalization of Fried’s phenotype.

The associations between objective measures of physical function and the study-specific mean of the SF-36 domain scores were weak. Dominant-handgrip strength did not show a significant correlation with the study-specific mean of the SF-36 domain scores (r = 0.064; *p* = 0.427). The same was true for the total SPPB score (r = 0.090; *p* = 0.267).

These results were maintained after multivariable adjustment. Handgrip strength did not show an independent association with the study-specific mean of the SF-36 domain scores (B = 0.080; β = 0.037; *p* = 0.660). The SPPB also did not show a statistically significant contribution (B = 0.451; β = 0.089; *p* = 0.307). When analyzing the physical function domain of the SF-36, a different pattern was observed. Handgrip strength was positively associated with that domain (r = 0.179; *p* = 0.026) and continued to show an independent relationship after adjusting for covariates (B = 0.452; β = 0.166; *p* = 0.041). In contrast, the SPPB did not show an independent relationship with perceived physical function (B = 0.412; β = 0.065; *p* = 0.440). The associations between handgrip strength, physical performance, and health-related quality of life are summarized in Table 2.

Multinomial logistic regression showed that the model including age, BMI, and history of falls was significantly associated with frailty status (χ^2^(8) = 26.233; *p* < 0.001). The pseudo R^2^ values were 0.156 for Cox-Snell, 0.226 for Nagelkerke, and 0.145 for McFadden. The likelihood ratio test confirmed that the study-specific mean of the SF-36 domain scores was associated with frailty status (χ^2^(2) = 12.945; *p* = 0.002).

When comparing robust participants with pre-frail participants, each 10-point increase in the study-specific mean of the SF-36 domain scores increased the odds of being in the robust group by 2.27 times (aOR = 2.27; 95% CI: 1.14–4.51; *p* = 0.018), regardless of age, BMI, or history of falls. Age and BMI did not show significant associations in this comparison.

When comparing frail participants with pre-frail participants, each 10-point increase in the study-specific mean of the SF-36 domain scores reduced the odds of being in the frail group by 44% (aOR = 0.56; 95% CI: 0.32–0.97; *p* = 0.040). In addition, a higher BMI reduced the odds of frailty compared with pre-frailty (aOR = 0.823; 95% CI: 0.703–0.963; *p* = 0.014). Age and history of falls did not show significant associations in this comparison.

A history of falls was significantly associated when comparing robust individuals with pre-frail individuals (aOR = 3.894; 95% CI: 1.178–12.870; *p* = 0.026), but the overall likelihood ratio test was not significant (*p* = 0.077), and the wide confidence interval indicated low precision. The results of the multinomial logistic regression analysis for frailty status are presented in Table 3.

## 4. Discussion

The main findings of the study reveal a high prevalence of pre-frailty in the evaluated population. In contrast, the prevalence of frailty and robustness was significantly lower. With regard to the SF-36, neither physical performance (SPPB) nor handgrip strength showed a significant association with the study-specific mean of the SF-36 domain scores. However, when specifically examining the dimension of perceived physical function in relation to handgrip strength, a significant correlation was found, whereas no such correlation was observed for the SPPB. Higher values of the study-specific mean of the SF-36 domain scores were associated with greater odds of being classified as robust and lower odds of being classified as frail, compared with the pre-frail category.

The results show a high prevalence of pre-frailty (82.6%). This finding should be interpreted in the context of the wide variation reported in other populations. A systematic review of South American studies reported a mean pre-frailty prevalence of 46.8%, reaching 50.7% among hospitalized older adults [19]. Similarly, a systematic review and meta-analysis of community-dwelling older adults in low- and middle-income countries reported a pooled pre-frailty prevalence of 49.3%, with substantial variation across individual studies [20]. In contrast, Bieniek et al. [21] found that frailty predominated among geriatric inpatients, accounting for 54.2%, whereas pre-frailty accounted for 35.8% (95% CI: 31.6–40.0).

Although this study was conducted exclusively among older adults in peri-urban communities of Montería, Colombia, some of the patterns observed are consistent with international evidence that has documented associations between frailty, functional decline, reduced muscle strength, and poorer health-related outcomes in older adults. This consistency suggests that the findings may share characteristics with those observed in other populations; however, it does not imply that the estimates obtained in this study are directly extrapolatable to other contexts. Cultural, socioeconomic, ethnic, and environmental differences, as well as differences in access to health services and in the criteria used to operationalize frailty, may alter both its prevalence and the magnitude of the observed associations. Therefore, the applicability of these results to populations in other countries and contexts should be considered with caution and requires confirmation through multicenter studies and more representative samples.

With regard to health-related quality of life (SF-36), its variation is multifactorial; therefore, a physical variable may be associated with greater or lesser intensity across the domains. Our research reveals a significant correlation between handgrip strength and the physical function domain. Meng et al. [14] reported a dose–response relationship between grip strength and quality of life. Similarly, Téllez et al. [22] reported a direct relationship between handgrip strength and functional and physical capacity in older adults. In another clinical population, Li et al. [23] found that handgrip strength was independently associated with the physical component of quality of life. Yoo et al. [15] also reported sex-specific associations, with lower handgrip strength being significantly associated with poorer HRQoL, particularly among older women. These findings support the view that handgrip strength is more closely related to physical aspects of perceived health than to all dimensions of overall quality of life.

Physical performance, as measured by the SPPB, can be assessed through a rapid, objective, and clinically applicable test [24]. However, when analyzing its relationship with physical function and overall quality of life, the SPPB did not show an independent association in the present study. Nevertheless, these results may be clinically relevant, since longitudinal evidence indicates that the SPPB can identify older adults at increased risk of adverse health outcomes and functional limitations [12]. Consequently, the Short Physical Performance Battery (SPPB) may be considered a complementary measure, rather than an equivalent one, to self-reports of physical function and quality of life.

Finally, our findings have relevant clinical implications, as they underscore the importance of a geriatric assessment that integrates objective measures of physical function, quality of life, limitations in activities, and risk of disability, as well as the monitoring of frailty using a variety of instruments. The high prevalence of pre-frail older adults in our study highlights the importance of early identification of frailty and pre-frailty [25]. Evidence from systematic reviews also supports non-pharmacological interventions, particularly physical activity-based approaches, as strategies that may help prevent progression or promote reversal of physical frailty [26].

Pre-frailty was the most prevalent category in the studied population of older adults. Grip strength showed a positive and independent association with the physical function domain of the SF-36. In contrast, physical performance assessed using the SPPB showed no independent association with either the study-specific mean of the SF-36 domain scores or with the physical function domain. Similarly, grip strength did not show an independent association with the study-specific mean of the SF-36 domain scores. These findings suggest that muscle strength may be more specifically related to the perception of one’s ability to perform physical activities than to a composite measure of health-related quality of life.

Furthermore, the results of the multinomial logistic regression model should be interpreted with caution due to the small number of participants in the “robust” (*n* = 14) and “frail” (*n* = 13) categories, compared with the “pre-frail” category (*n* = 128). This imbalance among the outcome categories may have reduced the accuracy and stability of the estimates, as reflected in the width of some confidence intervals. Consequently, the findings derived from the multinomial model should be considered exploratory and require confirmation in studies with larger samples and a more balanced distribution across the frailty categories.

This study has several limitations that should be considered when interpreting the results. First, participants were selected using non-probability convenience sampling in peri-urban communities in Montería, Colombia. Consequently, the sample cannot be considered representative of the general older adult population in Colombia or of older adult populations in other countries. Generalizations of the findings should be made with caution, especially in populations with sociodemographic, cultural, ethnic, economic, or health-care access characteristics that differ from those of the study population. Future multicenter studies, using probabilistic sampling strategies and including urban, rural, and geographically diverse populations, would allow for an assessment of the reproducibility and external validity of these findings. Second, there was a marked predominance of women (81.9%), which may limit the generalizability of the findings to older men. Gender-related differences in muscle strength, physical performance, and perceived quality of life could influence the observed associations. However, the small number of men included in this study (*n* = 28) limited the ability to conduct analyses stratified by sex with sufficient statistical precision.

Therefore, future studies should include samples with a more balanced distribution of men and women to specifically explore possible sex-related differences in the associations between physical performance, frailty, and health-related quality of life. Furthermore, the distribution of participants across frailty categories was markedly uneven, with only 14 participants classified as robust and 13 as frail, compared with 128 pre-frail participants. This imbalance may reduce the accuracy and stability of the estimates obtained through multinomial logistic regression; for this reason, the results derived from this analysis should be considered exploratory. Finally, due to the cross-sectional design of this study, it is not possible to establish temporal or causal relationships between physical performance, muscle strength, frailty status, and health-related quality of life.

## 5. Conclusions

In this population of peri-urban older adults, a high prevalence of pre-frailty (82.6%) was observed. Grip strength was positively and independently associated with the physical function domain of the SF-36, whereas no independent association was found between grip strength and the study-specific mean of the SF-36 domain scores. Physical performance assessed using the SPPB did not show any significant independent associations with either of these two measures.

Furthermore, a higher study-specific mean of the SF-36 domain scores was associated with a higher probability of being classified as robust and a lower probability of being classified as frail, compared with the pre-frail category. However, these results should be considered exploratory due to the small number of participants classified as robust and frail and the consequent risk of reduced precision and stability of the estimates.

Overall, these findings support the integrated assessment of muscle strength, physical performance, frailty, and health-related quality of life in older adults. Longitudinal studies with larger probability samples and a more balanced distribution across frailty categories are needed to confirm these associations and establish their temporal relationship.

## Figures and Tables

**Table 1:** Sociodemographic, clinical, functional, and quality-of-life characteristics of the participants.

Variable	Result
Age, years, mean ± SD	71.94 ± 6.63
Sex, *n* (%)	
Male	28 (18.1)
Female	127 (81.9)
Educational level, *n* (%)	
Low	50 (32.3)
Medium	53 (34.2)
High	52 (33.5)
BMI, kg/m^2^, mean ± SD	27.68 ± 4.57
Morbidity burden, *n* (%)	
Low burden	79 (51.0)
High burden	76 (49.0)
Polypharmacy, *n* (%)	
No	45 (29.0)
Yes	110 (71.0)
Falls during the previous year, *n* (%)	
Yes	41 (26.5)
No	114 (73.5)
Dominant handgrip strength, kg, mean ± SD	20.08 ± 5.13
Total SPPB, points, mean ± SD	7.23 ± 2.22
Study-specific mean of SF-36 domain scores, mean ± SD	57.02 ± 11.29
SF-36 Physical Functioning, mean ± SD	50.04 ± 13.96
Fried’s Frailty Status, *n* (%)	
Robust	14 (9.0)
Pre-frail	128 (82.6)
Frail	13 (8.4)

Note: SD = standard deviation; BMI = Body Mass Index; SPPB = Short Physical Performance Battery; SF-36 = 36-Item Short Form Health Survey. N = 155.

**Table 2:** Association of handgrip strength and physical performance with health-related quality of life.

Functional Variable	Outcome	r	Bivariate *p*-Value	Adjusted B (95% CI)	Adjusted β	Adjusted *p*
Dominant handgrip strength	Study-specific mean of SF-36 domain scores	0.064	0.427	0.080 (−0.280 to 0.441)	0.037	0.660
Total SPPB	Study-specific mean of SF-36 domain scores	0.090	0.267	0.451 (−0.418 to 1.320)	0.089	0.307
Dominant handgrip strength	SF-36 Physical Functioning	0.179	0.026	0.452 (0.019 to 0.886)	0.166	0.041
Total SPPB	SF-36 Physical Functioning	0.066	0.414	0.412 (−0.638 to 1.461)	0.065	0.440

Note: r = Pearson correlation coefficient; B = unstandardized regression coefficient; 95% CI = 95% confidence interval; β = standardized coefficient. The linear models were adjusted for age, sex, BMI, education level, multimorbidity, and polypharmacy.

**Table 3:** Multinomial logistic regression adjusted for the association between quality of life and frailty level.

Predictor	Robust vs. Pre-Frail aOR (95%CI)	*p*	Frail vs. Pre-Frail aOR (95%CI)	*p*
Study-specific mean of SF-36 domain scores, +10 points	2.27 (1.14–4.51)	0.018	0.56 (0.32–0.97)	0.040
Age, +1 year	1.028 (0.940–1.125)	0.546	1.034 (0.941–1.136)	0.489
BMI, +1 kg/m^2^	0.952 (0.828–1.094)	0.487	0.823 (0.703–0.963)	0.014
Falls, yes vs. no	3.894 (1.178–12.870)	0.026	1.577 (0.435–5.713)	0.489

Note. aOR = adjusted odds ratio; 95% CI = 95% confidence interval; BMI = body mass index; SF-36 = 36-Item Short Form Health Survey. Reference category for the dependent variable: pre-frail. Overall model: χ^2^ (8) = 26.233; *p* < 0.001; Nagelkerke R^2^ = 0.226.

## Data Availability

The data presented in this study are available on request from the corresponding author due to ethical and privacy restrictions related to the protection of participants’ confidential information.

## Abbreviations

The following abbreviations are used in this manuscript:

SF36	Short Form-36 Health Survey
SPPB	Short Physical Performance Battery
HRQoL	Health-Related Quality of Life
